# Dietary phytosterols improves the metabolic status of perinatal cows as evidenced by plasma metabolomics and faecal microbial metabolism

**DOI:** 10.5713/ab.23.0422

**Published:** 2024-04-26

**Authors:** Jian Gao, Donghai Lv, Zichen Wu, Zhanying Sun, Xiaoni Sun, Suozhu Liu, Zhankun Tan, Weiyun Zhu, Yanfen Cheng

**Affiliations:** 1 Laboratory of Gastrointestinal Microbiology, National Centre for International Research on Animal Gut Nutrition, Nanjing Agricultural University, Nanjing 210095, China; 2 College of Animal Science, Tibet Agricultural and Husbandry University, Nyingchi 860000, China

**Keywords:** Faecal Microbe, Metabolic Status, Perinatal Cow, Phytosterols, Plasma Metabolome

## Abstract

**Objective:**

Previous research reported that dietary addition with phytosterols improved the energy utilisation of the rumen microbiome, suggesting its potential to alleviate the negative energy balance of perinatal cows. This experiment aimed to explore the effects of feeding phytosterols on the metabolic status of perinatal cows through plasma metabolomics and faecal bacteria metabolism.

**Methods:**

Ten perinatal Holstein cows (multiparous, 2 parities) with a similar calving date were selected four weeks before calving. After 7 days for adaptation, cows were allocated to two groups (n = 5), which respectively received the basal rations supplementing commercial phytosterols at 0 and 200 mg/d during a 42-day experiment. The milk yield of each cow was recorded daily after calving. On days 1 and 42, blood and faeces samples were all collected from perinatal cows before morning feeding for analysing plasma biochemicals and metabolome, and faecal bacteria metabolism.

**Results:**

Dietary addition with phytosterols at 200 mg/d had no effects on plasma cholesterol and numerically increased milk yield by 1.82 kg/d (p>0.10) but attenuated their negative energy balance in perinatal cows as observed from the significantly decreased plasma level of β-hydroxybutyric acid (p = 0.002). Dietary addition with phytosterols significantly altered 12 and 15 metabolites (p<0.05) within the plasma and faeces of perinatal cows, respectively. Of these metabolites, 5 upregulated plasma fatty acids indicated an improved energy status (i.e., C18:1T, C14:0, C17:0, C18:0, and C16:0). Milk yield negatively correlated with plasma concentrations of ketone bodies (p = 0.035) and 5-methoxytryptamine (p = 0.039). Furthermore, dietary addition with phytosterols at 200 mg/d had no effects on fermentation characteristics and bacterial diversity of cow faeces (p>0.10) but improved potentially beneficial bacteria such as Christensenellaceae family (p<0.05) that positively correlated with feed efficiency.

**Conclusion:**

Dietary addition with phytosterols at 200 mg/d could effectively improve the energy status in perinatal cows by attenuating their negative energy balance.

## INTRODUCTION

Phytosterols are bioactive compounds in plants, especially in oilseeds. Due to similar structures, phytosterols compete with cholesterols for the incorporation into mixed micelles and could inhibit intestinal absorption or alter the metabolism pathway of cholesterols in the body of animals [1]. It is not surprising that studies reported dietary phytosterols significantly decreased the blood concentrations of cholesterols and lipoprotein cholesterols in rodents [2]. Besides, phytosterols also have other beneficial functions for animal health, such as inflammatory cytokine inhibition [3], modification of blood lipid profiles and increased activity of thyroid glands [4]. Phytosterols are also favourable for ruminants, but related reports are still few. A previous study reported that adding phytosterols at a 30 mg/kg ration improved nutrient digestibility and microbial protein synthesis of cows’ rumen microbiota *in vitro* [5]. Their findings could explain the results of Xie et al [6], who found supplementing phytosterols at 200 mg/d increased the milk yield by 1.71 kg/d and decreased the blood cholesterol levels in dairy cows. Lv et al [7] further reported that adding 200 mg phytosterols/d improved propionate synthesis and microbial growth of rumen microbiota in perinatal cows by modifying the active rumen microbiome. The above results indicated that dietary phytosterols would promote energy utilisation in dairy cows by improving rumen fermentation.

During the perinatal period, cows suffer acute stresses due to the increased demand for energy, decreased feed intake, hormone changes, and others [8]. These risks would aggravate the negative energy balance and further result in metabolic disorders in perinatal cows, such as ketosis and fatty liver. Our laboratory has reported that supplementing phytosterols at 200 mg/d could improve energy utilisation, especially propionate synthesis, and growth of the rumen microbiome in perinatal cows [7]. Ruminal propionate is an energy precursor for gluconeogenesis and could decrease fatty acid oxidisation in the liver of cows [9]. Thus, the increased propionate by dietary phytosterols would also depress the decomposition of body fat and hepatic dysfunctions caused by the negative energy balance in cows. However, it is still unclear what are the actual effects of supplementing phytosterols to attenuate the negative energy balance in cows. Here, we first determine the blood indices of cows since some blood chemicals such as β-hydroxybutyric acid (BHBA) [10] are indicators for energy status. Besides, metabolite profiles in different biofluids obtained by metabolomics are efficient tools for evaluating health and nutrient metabolism in ruminants [11,12]. The present experiment aimed to investigate the changes in the metabolites of plasma and faeces in response to supplementing phytosterols in perinatal cows. Given the low intestinal absorption of phytosterols (only 0.4% to 3.5%) [13], whether phytosterols affect the bacterial community in the faeces of cows is also reported in this experiment, because the hindgut microbiota also provides about 12% of energy precursors for ruminants [14] and plays a role in host health [15]. The results of this experiment could provide new insights into the bioactive functions of phytosterols for the health status and metabolism of ruminants.

## MATERIALS AND METHODS

### Ethics approval

The use of animals in this experiment was approved by the Animal Care and Use Committee of Nanjing Agricultural University in Nanjing, Jiangsu, China (SYXK [SU] 2017-0007).

### Experimental design, animal feeding and sampling

Ten perinatal Holstein cows (multiparous, 2 parities) with a similar calving date were selected as the experimental animals within a cohort of more than 50 perinatal cows four weeks before calving. After one week for adaptation, the experimental animals were allocated to two groups (n = 5), which respectively received commercial dry powders of phytosterols at 0 and 200 mg/d once at morning feeding during a 6-week experiment as described by Lv et al [7]. Dry powders of phytosterols were directly fed to the cows once in the morning feeding. The additional level of phytosterols is according to Xie et al [6] who found supplementing phytosterols at 200 mg/d increased the milk yield by 1.71 kg/d in dairy cows at late lactation. Phytosterols (purity 91.14%) used in this experiment were from Nanjing Nature Bio-Tech Co., Ltd. (Jiangsu, China) and mainly comprised 44.71% sitosterol, 27.23% campesterol, and 16.63% stigmasterol. The experiment lasted 21 days before and after the expected calving date. Total mixed rations (Table 1) were delivered to the perinatal cows at 07:00 and 17:00 daily. Each cow was kept in a separate pen and had free access to diets and drinking water. On days 1 and 42, blood and faeces samples were all collected from perinatal cows before feeding in the morning. Blood samples from each cow were taken from the jugular vein using vacutainers (BD Medical Instrument Co., Ltd, Shanghai, China), while rectal faeces samples were collected and then dispensed into sterile tubes. The cows’ blood was centrifuged at 2,000 g for 15 min and the supernatant plasma samples retained. After calving, the cows were milked three times a day (04:30, 12:30, 20:30) and the daily milk yields of each cow recorded. All samples were kept at −80°C for later analysis.

### Analysis of plasma indices

Plasma concentrations of acetoacetic acid and acetone were determined using ELISA kits for bovine (Beijing Fangcheng Biotechnology Co., Ltd., Beijing, China) and an RT-6100 microplate reader (Rayto Life and Analytical Sciences Co., Ltd., Shenzhen, China). Plasma BHBA was analysed using the commercial kit (no. E030-1-1) from Nanjing Jiancheng Bioengineering Institute (Nanjing, China). The total plasma concentration of ketone bodies was obtained by summing the concentrations of acetoacetic acid, acetone and BHBA. Plasma concentrations of glucose, triglyceride, total cholesterol, and lipoprotein cholesterols were determined using an A6 Biochemical Analyser (Beijing Shining Sun Technology Co., Ltd, Beijing, China) and kits from BioSino Bio-Technology & Science Inc. (Beijing, China).

### Extraction and Identification of metabolites using GC-TOF-MS

The metabolites in plasma and faecal samples were extracted according to the methods described in detail by Gao et al [11]. Briefly, metabolites were extracted using a methanol solution and mixed with L-2-chlorophenylalanine solution (an internal standard). The mixture was centrifuged at 17,000 g for 15 min. An equal volume of each supernatant was pooled into the quality control (QC) samples for plasma or faeces. The remaining supernatant from each sample was dried individually using a vacuum concentrator. Next, dried extracts of samples were incubated with methoxyamination hydrochloride at 80°C for 30 min and derived with bis(trimethylsilyl)trifluoroacetamide solution containing 1% trimethylchlorosilane (v/v) at 70°C for 1.5 h. The metabolites in the derived samples were subsequently determined using the 7890A gas chromatograph (Agilent Technologies, Santa Clara, CA, USA) coupled with a Pegasus HT time-of-flight mass spectrometer (GC-TOF-MS; LECO Corporation, MI, USA) following the test conditions described by Sun et al [12]. Raw peaks of metabolomics were pre-treated using the ChromaTOF software (version 4.3x; LECO Corporation, Saint Joseph, MI, USA) and annotated with the LECO-Fiehn Rtx5 database. Peak areas were normalized using the internal standard within each sample. Peaks were removed if relative standard deviations of peak area in QC samples exceeded 30% or less than 50% of total samples. Missing values of each metabolite were estimated using the method of K-Nearest Neighbour.

### Faecal fermentation characteristics

Faecal samples were mixed with distilled water at a ratio of 1:4 (w/v) in tubes. The pH values of the mixture were measured using a portable pH meter immediately after mixing. Then, faecal mixtures were centrifuged at 3,000 g for 15 min to obtain the liquid supernatant for analysing fermentation characteristics. Ammonia-N concentration was analysed using a colourimetry method according to Broderick and Kang [16]. The concentration of microbial crude protein was determined using the method of Makkar et al [17] with Coomassie blue solution and an RT-6100 microplate reader (Rayto Life and Analytical Sciences Co., Ltd., Shenzhen, China). Concentrations of volatile fatty acids were determined using a 7890B gas chromatograph (Agilent Technologies, USA) equipped with a fused silica capillary column (Supelco, Bellefonte, PA, USA) as described by Jin et al [18]. The contents of ammonia-N, microbial crude protein, and volatile fatty acids in faecal samples were obtained by their concentrations in mixtures of faeces and water multiplied by the dissolution ratio (1:4, w/v).

### 16S rRNA sequencing of the bacterial community in cows’ faeces

Genomic DNA of faecal microbiota was obtained by the CTAB method [19] and the concentration and quality were determined with a Nanodrop 2000 UV-vis spectrophotometer (Thermo Fisher Scientific, Sunnyvale, DE, USA). The primers 515F (GTGCCAGCMGCCGCGGTAA) and 806R (GGAC TACHVGGGTWTCTAAT) were used for the target amplifications in the V4 region of bacterial 16S rRNA. The polymerase chain reaction (PCR) products were purified using the Agencourt AMPure XP kit (Beckman Coulter Trading Co., Ltd., Shanghai, China) and sequenced on the HiSeq platform (Illumina, Madison, WI, USA) after library construction. All sequencing procedures were performed by the Beijing Genomics Institute (China). Raw sequences were respectively filtered and pre-treated according to Fadrosh et al [20] to remove the low-quality reads. Clean reads were clustered at 99% similarity to obtain the operational taxonomic units (OTU). Chimaera formed during PCR amplification were deleted from the OTU data using the UCHIME software (version 4.2.40). The SILVA database (138.1) was referred to annotate faecal bacteria.

### Statistical analysis

Results of blood indices and faecal parameters were analysed using the one-way analysis of covariance with R software (version 4.0.3) with the initial data on the first day as the covariates. The data were deemed significant if p<0.05 and as tendencies if 0.05<p<0.10. All data were listed as least-squares mean±pooled standard error of means. For metabolome analysis, both the principal component analysis and orthogonal partial least-squares discrimination analysis (OPLS-DA) of metabolites in plasma or faeces were conducted using the MetaboAnalyst (version 5.0). Differential metabolites between treatments were identified using the variable importance in projection (VIP) obtained from the OPLS-DA and p-values from the student’s t-test (VIP>1, p<0.05). The fold change (FC) for each metabolite was calculated by the relative concentration of treatments divided by that of the control. The relationships of milk yield and blood biochemicals to biofluid metabolites were analysed using the Spearman rank correlation and visualised using Gephi (version 0.10.1). The community diversity of faecal bacteria was analysed using QIIME2 containing indices for alpha diversity and the principal coordinates analysis. Differential bacteria between treatments were identified using linear discriminant analysis (LDA) effect size (LEfSe) [21] based on LDA>2 and p<0.05.

## RESULTS

### Milk yield and plasma chemicals in cows

Table 2 shows that supplementing phytosterols at 200 mg/d had no effect on dry matter intake (p>0.10) and only numerically increased the daily milk yield by 1.82 kg/d (p>0.10) of cows during the perinatal period. For plasma chemicals, supplementing phytosterols significantly decreased the plasma concentration of BHBA (p = 0.002) and increased that of acetoacetic acid (p = 0.002; Table 2). It also tended to increase plasma triglyceride concentration (p = 0.074) but had no effects on the plasma concentrations of glucose, total cholesterol, acetone, and lipoprotein cholesterols in postpartum cows (p>0.10).

**Figure 1:**
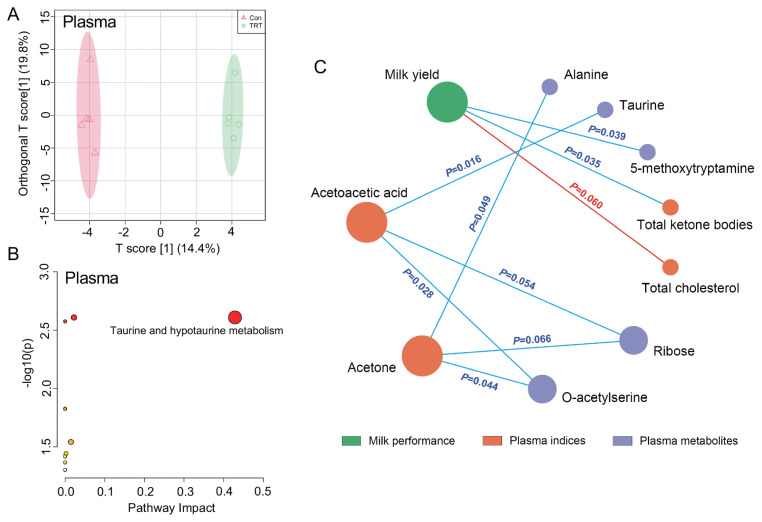
Effects of dietary addition of phytosterol on the plasma metabolites of postpartum cows and their correlations with milk yield. (A) Orthogonal partial least-squares discrimination analysis (OPLS-DA) analysis of plasma metabolites. (B) Pathway impact based on the differential plasma metabolites. (C) Associated network of milk yield with plasma indices and plasma differential metabolites caused by phytosterols using the analysis of Spearman rank correlation (│coefficient│<0.6, p<0.10).

A total of 129 plasma metabolites were identified in the present experiment. Results of OPLS-DA showed that supplementing phytosterols at 200 mg/d caused a clear separation in plasma metabolites (Figure 1A). Supplementing phytosterols at 200 mg/d upregulated the relative concentrations of 6 plasma metabolites containing elaidic acid, myristic acid, heptadecanoic acid, stearic acid, atrazine-2-hydroxy, and palmitic acid (FC>1.40, p<0.05; Table 3). It also downregulated seven plasma metabolites containing glycolic acid, O-acetylserine, alanine, taurine, ribose, inosine, and 5-methoxytryptamine (FC<0.87, p<0.05). The result of pathway impact indicated that only the taurine and hypotaurine metabolism pathway was enriched based on these differential plasma metabolites (Figure 1B).

Figure 1C shows that the milk yield had negative correlations with plasma concentration of total ketone bodies (R = −0.685, p = 0.035) and 5-methoxytryptamine (R = −0.673, p = 0.039) but tended to have a positive correlation with plasma cholesterol (R = 0.624, p = 0.060). Both plasma concentrations of acetoacetic acid and acetone significantly negatively correlated with O-acetylserine (R = −0.709, p = 0.028; R = −0.661, p = 0.044) and tended to have negative correlations with plasma ribose (R = −0.636, p = 0.054; R = −0.612, p = 0.066). The concentration of acetoacetic acid also negatively correlated with that of taurine (R = −0.758, p = 0.016), while the acetone concentration showed a negative correlation with that of alanine (R = −0.636, p = 0.049) in the perinatal cows.

### Bacterial community in faeces of cows

**Figure 2:**
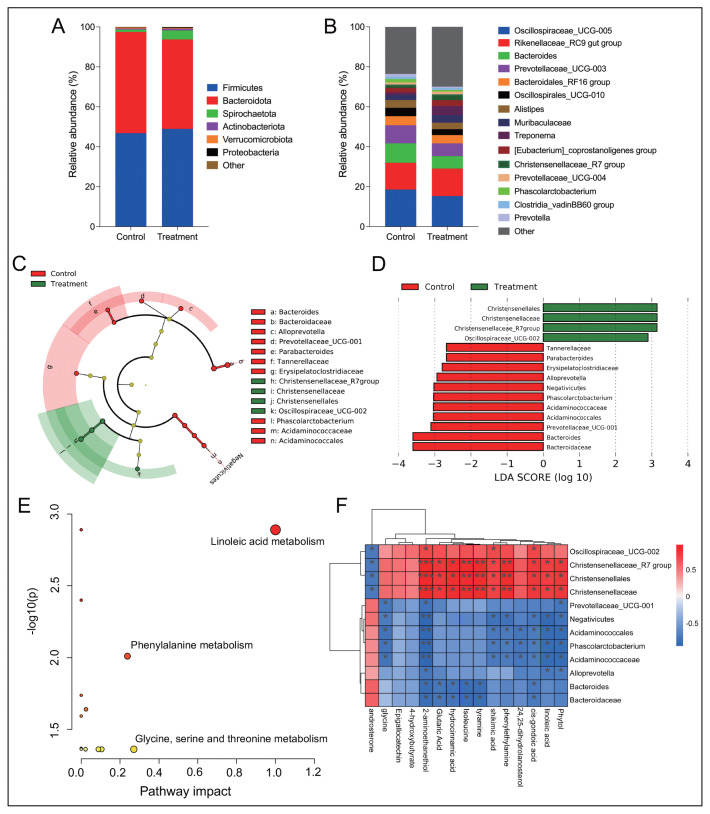
Effects of dietary addition of phytosterol on the faecal bacterial community of postpartum cows. (A, B) Major bacteria at phylum and genus levels. (C, D) Differential bacteria in the faeces of postpartum cows fed phytosterol or not using LEfSe analysis (LDA>2, p<0.05). (E) Enrich pathways based on the differential metabolites in the faeces of postpartum cows (FDR<0.05). (F) Relationships between differential faecal bacteria and differential faecal metabolites (R>0.6, FDR<0.05). LDA, linear discriminant analysis; LEfSe, linear discriminant analysis effect size; FDR, false discovery rate.

Table 4 shows that supplementing phytosterols at 200 mg/d had no effect on the alpha diversity of the bacterial community in the faeces of cows (p>0.10). Firmicutes and Bacteroidota were the dominant bacterial phyla in the faeces of cows (Figure 2A), while the most abundant bacterial genera were *Oscillospiraceae_UCG-005*, *Rikenellaceae_RC9 gut group*, *Bacteroides and Prevotellaceae_UCG-003* (Figure 2B). Supplementing phytosterols at 200 mg/d significantly enriched the relative abundances of Christensenellales, Christensenellaceae, *Christensenellaceae_R7 group* and *Oscillospiraceae_UCG-002* (LDA>2, p<0.05; Figure 2C and 2D), but decreased the relative abundances of Negativicutes, Acidaminococcales, Tannerellaceae, Erysipelatoclostridiaceae, Acidaminococcaceae, Bacteroidaceae, *Parabacteroides*, *Alloprevotella*, *Phascolarctobacterium*, *Prevotellaceae_UCG-001*, and *Bacteroides* (LDA >2, p<0.05).

### Characteristics and metabolites in faeces of cows

Table 5 shows that supplementing phytosterols at 200 mg/d had no effect on the fermentation characteristics in the faeces of cows containing the pH value, concentrations of ammonia-N, lactate, microbial crude protein, and volatile fatty acids (p>0.10). However, it could upregulate 14 metabolites in cow faeces, which were isoleucine, 4-hydroxybutyrate, 2-aminoethanethiol, tyramine, phenylethylamine, hydrocinnamic acid, linoleic acid, glutaric acid, epigallocatechin, cis-gondoic acid, 24,25-dihydrolanosterol, phytol, glycine, and shikimic acid (FC>1.38, p<0.05; Table 6). Supplementing phytosterols downregulated only one faecal metabolite named androsterone (FC = 0.64, p = 0.043). Based on the differential metabolites in faeces, the results of pathway impact indicated that supplementing phytosterols could enrich the pathways of linoleic acid metabolism, phenylalanine metabolism, and glycine, serine and threonine metabolism (p<0.01, pathway impact>0.2; Figure 2E). Results of spearman analysis showed that Christensenellales, Christensenellaceae, *Oscillospiraceae_UCG-002*, and *Christensenellaceae_R7 group* had significant correlations with most differential faecal metabolites (p<0.05), while the rest of differential bacteria showed negative correlations with these metabolites (Figure 2F).

## DISCUSSION

Phytosterols are well-known as cholesterol-reducing compounds for nonruminants [2]. Current studies reported that phytosterols could reduce blood cholesterol by inhibiting intestinal absorption of cholesterol or affecting cholesterol metabolism in the host [1]. However, in the present experiment, dietary phytosterols did not affect the plasma concentrations of total cholesterol and lipoprotein cholesterol in cows. The reason could mainly be two aspects based on the ways phytosterols act. One aspect is that phytosterols could not effectively affect the intestinal absorption of cholesterols in ruminants since dietary cholesterols are mainly from animal foodstuff rather than the plant feeds fed to ruminants [22]. The other aspect is that phytosterols were degraded by gastrointestinal microbes [23]. Cows usually suffer a negative energy balance due to the transition of their physical status. Energy deficiency would increase the metabolism and mobilisation of body fat and increase the concentration of non-esterified fatty acids in perinatal cows to acquire the energy demand for the hosts. Normally, farmers would add fats into the cows’ diet after calving, which incurs additional feed costs, and excess dietary fat disturbs the activities and fermentation of ruminal microbes [24]. Previous studies reported that dietary phytosterols could modify the rumen bacterial community to maintain the health of dairy goats fed high-grain diets [25] and a low dose of phytosterols enriched ruminal glucoside hydrolase family 13 for starch degradation and that specific bacteria such as *Fibrobacter succinogenes* were enriched in the rumen of feed-efficient cattle to improve the energy status of cows [7,26]. These results indicated that the low dose of phytosterols required less additional feed cost for attenuating the negative energy balance of cows and induced growth-promoting effects on rumen microbes compared with adding fat to the diets of ruminants. Moreover, in the present experiment, dietary phytosterols did not alter the plasma glucose in perinatal cows, though it could increase the production of blood glucose precursors (ruminal propionate) [7]. This result agreed with Salehi-Sahlabadi et al [27], who found that phytosterol ingestion did not affect human blood glucose. The reason could be that blood glucose is an insensitive marker of energy status in cows [28].

Blood ketone bodies, especially BHBA in cow blood, are indicators of the negative energy balance because excess non-esterified fatty acids (NEFA) are not completely degraded in the liver but oxidised into BHBA [8]. Blood BHBA concentration could also be the only predictor in the mathematical model for predicting the negative energy balance in cows [10]. In the present experiment, dietary phytosterols significantly decreased the plasma BHBA from 1.18 to 0.63 mmol/L, suggesting greater energy utilisation in cows. For another, the plasma BHBA concentration in the control group is extremely close to the lowest limitation for postpartum hyperketonemia (>1.2 mmol/L) [29]. A large-scale study with 2,758 cows reported that post-perinatal cows with a blood BHBA concentration >1.0 mmol/L had more than 4 times higher risk of postpartum diseases such as metritis and clinical ketosis [30]. Thus, dietary phytosterols not only had the potential to attenuate the negative energy balance but also decrease the risk of postpartum diseases in cows. However, the results of the present experiment also showed that dietary phytosterols increased the plasma concentration of acetoacetic acid but had no effects on plasma acetone and total ketone bodies. A previous study reported that acetyl-CoA is the precursor for ketogenesis to produce acetate, acetoacetate, and BHBA in the liver of cows [31]. Their results suggested that dietary phytosterols would not affect the utilisation rate of acetyl-CoA but alter its metabolic pathway in the liver. Unlike rodents, the hepatic cells of ruminants could not reduce acetoacetate to BHBA due to the lack of BHBA dehydrogenase [32]. The acetoacetate within the mitochondria could be exchanged with pyruvate from the cytosol to increase energy utilisation and pyruvate uptake in hepatic cells [32]. This result could be the reason for the increased plasma acetoacetate in cows fed phytosterols because we previously found that pyruvate was enriched in the rumen of cows fed phytosterols [7] while its relative concentration tended to decrease in the plasma in the present experiment. Bergseth et al [33] reported that the decrease of phytosterols in soybean oil by the hydrogenated treatment could improve the ketogenesis from low-chain fatty acids in isolated hepatocytes of rats. However, the specific mechanism still needs to be investigated using the hepatic cells of ruminants *in vitro*.

The NEFA is also an indicator that showed the same variation trend with blood BHBA in cows suffering from negative energy balance, while changes in the blood concentrations of C18:1ω9, C18:1ω7, and C18:3ω3 are most predictive for negative energy balance based on a large-scale experiment [34]. In our study, five plasma free fatty acids, elaidic acid (C18:1T), myristic acid (C14:0), heptadecanoic acid (C17:0), stearic acid (C18:0) and palmitic acid (C16:0), were upregulated by dietary phytosterols. C17:0 is a free fatty acid derived from plant oil, while C14:0 (<1%) and C18:1T (about 1% to 2%) are quite low in the fatty acid composition of cows [35]. Furthermore, Douglas et al [35] reported that the percentage of free C14:0, C16:0, and C18:0 in plasma decreased in the cows with negative energy balance following calving while the plasma *Cis*-18:1, the major fatty acid in adipose tissue (45.01% to 50.54%), increased. Rukkwamsuk et al [36] reported that restricted energy intake in cows after parturition significantly decreased the plasma concentrations of C16:0, C18:0, cis C18:1 with numerical decreases in C14:0 and C18:1T. Their results indicated that the increase of these plasma fatty acids in the present experiment resulted in an improved energy status in the cows rather than derived from the negative energy balance, which simultaneously increases plasma concentrations of BHBA and NEFA [34]. The C16:0 and C18:0 fatty acids also have other functional roles in cows such as milk fat synthesis in the mammary gland [37] rather than just being an energy source. Besides, dietary phytosterols downregulated the other 7 plasma metabolites and increased the taurine and hypotaurine metabolism pathway. Taurine is a non-protein sulphur-containing amino acid, and endogenous taurine in the blood was derived from cystine and methionine in the liver of animals [38]. The reason for decreased plasma taurine could be because dietary phytosterols enhance the rumen microbiome’s cysteine and methionine metabolism pathway and improve microbial protein synthesis [7].

Although our previous finding showed that supplementing phytosterols at 200 mg/d could improve rumen energy utilisation in perinatal cows [7], results of the present experiment indicated that dietary supplement with phytosterols showed no significant influences on milk yield of post-perinatal cows. This result disagreed with Xie et al [6] reported supplementing phytosterols at 200 mg/d significantly increased the milk yield by 1.71 kg/d in dairy cows at late lactation. The reason could be that the increased energy utilisation was not only used for milk lactose synthesis and milk production but also for the recovery of perinatal cows from the negative energy balance, which was observed from the negative correlation between milk yield and plasma concentration of ketone bodies in the present experiment. Besides, only one differential metabolite (i.e., 5-methoxy tryptamine) in cow biofluids caused by phytosterols significantly correlated with milk yield. 5-methoxy tryptamine is an alternative precursor for melatonin synthesis, and the injection of 5-methoxy tryptamine could increase plasma melatonin in rats [39]. However, increased plasma melatonin levels depressed the milk yield in cows given melatonin implants [40]. The above results could explain the negative correlation between plasma 5-methoxy tryptamine and milk yield in the present experiment. Results also showed that plasma concentrations of ketone bodies showed negative correlations with O-acetylserine, taurine, and alanine suggesting these amino acids had the potential to affect lipolysis in perinatal cows. However, actual mechanisms are still unclear and need to be further studied.

Given the microbial conversion and intestinal absorption of phytosterols in animals [13,23], the low amount of phytosterols would not directly affect the fermentation and community structure of faecal microbiota in cows in the present experiment. Not surprisingly, the results of our study found that dietary phytosterols did not affect the fermentation characteristics and alpha diversity of faecal bacteria in perinatal cows. Interestingly, we found that the phytosterols altered the relative abundances of faecal bacteria and their metabolites, partly because the alteration of forestomach digestion would change the digesta composition flowed to the hindgut and subsequently the bacterial network in ruminant faeces [41]. Firmicutes and Bacteroidota were the major bacterial phylum in the faeces of cows. In the present experiment, dietary phytosterols only increased the relative abundances of Christensenellaceae and Oscillospiraceae families. Both Christensenellaceae and Oscillospiraceae species are butyrate-producing bacteria in the digestive tract of animals, which help maintain intestinal structure and functions [42]. In addition, previous studies reported that the Christensenellaceae family was more abundant in the faeces of cattle with a high feed conversion rate [43]. Thus, dietary phytosterols have the potential to improve hindgut health and feed efficiency in cows.

## CONCLUSION

Dietary addition with phytosterols at 200 mg/d numerically increased the milk yield by 1.82 kg/d in perinatal cows. It did not affect plasma cholesterol metabolism in perinatal cows but attenuated their negative energy balance observed from the significantly decreased plasma BHBA level. Besides, dietary addition with phytosterols upregulated five free fatty acids in cow plasma (i.e., C18:1T, C14:0, C17:0, C18:0, and C16:0), indicating an improvement in energy status in perinatal cows. Dietary addition with phytosterols at 200 mg/d could improve potentially beneficial bacteria such as Christensenellaceae, a bacterial family positively correlated with feed efficiency, though it did not affect the bacterial diversity within cow faeces. Summarily, dietary addition with phytosterols at 200 mg/d could effectively improve the energy status in perinatal cows to attenuate their negative energy balance. Additionally, further studies are still needed to evaluate a more suitable dose of dietary phytosterols in perinatal cows for both health recovery and milk yield.

## Figures and Tables

**Table 1:** Ingredients of the basal rations and their chemical compositions (% of dry matter except for net energy)

Items	Pre-calving	Post-calving
Ingredient		
Wheat straw	30.48	-
Oat hay	7.44	9.46
Alfalfa hay	-	10.88
Corn silage	16.82	27.51
Corn	15.18	11.72
Soybean meal	14.32	16.64
Steam flaked corn	-	5.43
Beet pulp	3.30	5.59
Wheat bran	3.17	-
Corn gluten meal	3.83	-
Cottonseed meal	-	3.97
Molasses	-	1.01
Fat powder	-	2.42
NaHCO3	1.05	1.10
CaPO3	0.56	0.94
NaCl	0.45	0.66
Choline chloride	0.40	-
Premix^1)^	3.00	2.67
Nutrient composition		
Net energy (MJ/kg)	5.18	5.88
Crude protein	12.38	16.43
Neutral detergent fibre	53.81	31.95
Acid detergent fibre	30.55	16.43
Non-fibre carbohydrate	24.75	40.02
Ether extract	1.88	5.04
Calcium	0.40	0.85
Phosphorus	0.25	0.38

1) One kg of premix contained 240 mg of vitamin A, 4.5 mg of vitamin D3, 5.4 g of vitamin E, 920.0 mg of vitamin B_1_, 1.2 g of vitamin B_2_, 10 of mg vitamin B_12_, 100.8 of mg biotin, 30.0 g nicotinic acid, 270.0 mg D-pantothenic acid, 1.0 g Fe, 1.3 g Mn, 1.8 g Zn, 180 mg I, 70 mg Se, 40 mg Co.

**Table 2:** Effects of supplementing phytosterols on the milk yield and plasma indices in perinatal cows

Items	Phytosterol level (mg/head/d)		SEM	p-value

	0	200		
Dry matter intake (kg/d)	12.95	13.42	0.538	0.554
Milk yield (kg/d)	33.75	35.57	2.068	0.553
Plasma indices (mmol/L)				
β-Hydroxybutyric acid	1.18	0.63	0.073	0.002
Acetoacetic acid	0.575	0.809	0.033	0.002
Acetone	0.423	0.409	0.022	0.697
Total ketone bodies	2.14	1.88	0.095	0.112
Glucose	3.04	3.20	0.245	0.669
Triglyceride	0.144	0.166	0.007	0.074
Total cholesterol	3.18	3.23	0.287	0.892
High-density lipoprotein cholesterol	1.76	1.78	0.132	0.915
Low-density lipoprotein cholesterol	0.997	0.995	0.108	0.993

All data were listed as least-squares mean±pooled standard error of means (SEM).

The data were deemed significant if p<0.05 and as tendencies, if 0.05<p<0.10.

**Table 3:** Differential metabolites in the plasma of perinatal cows fed with/without phytosterols

Compound	Similarity	Mass	VIP^1)^	FC^2)^	p-value
Elaidic acid (C18:1T)	904.4	117	1.78	3.51	0.025
Myristic acid (C14:0)	842.1	117	1.77	2.57	0.027
Heptadecanoic acid (C17:0)	856.5	117	1.71	2.09	0.035
Stearic acid (C18:0)	967.0	117	1.73	1.54	0.034
Palmitic acid (C16:0)	969.6	117	1.62	1.40	0.049
Glycolic acid	873.9	66	1.67	0.87	0.046
O-acetylserine	223.7	116	1.74	0.80	0.044
Alanine	916.3	116	1.93	0.63	0.015
Taurine	781.9	59	2.23	0.57	0.002
Ribose	714.6	103	2.19	0.54	0.003
Inosine	749.2	73	1.77	0.33	0.036
5-Methoxytryptamine	785.5	174	1.73	0.19	0.038

1) Variable importance in projection (VIP) was obtained from orthogonal partial least-squares discrimination analysis of metabolites.

2) Fold change (FC) was calculated through the peak area of each metabolite in the treatment group divided by the peak area of the corresponding metabolite in the control group.

**Table 4:** Effects of supplementing phytosterols on the alpha diversity of faecal bacteria in perinatal cows

Items	Phytosterols added (mg/d)		p-value

	0	200	
Sobs (×10^3^)	0.87±0.03	0.95±0.14	0.302
Chao (×10^3^)	1.03±0.07	1.06±0.16	0.755
Ace (×10^3^)	1.00±0.05	1.05±0.15	0.524
Shannon	4.93±0.04	5.03±0.31	0.501

All data were listed as least-squares mean±standard deviation.

The data were deemed significant if p<0.05 and as tendencies, if 0.05<p< 0.10.

**Table 5:** Effects of supplementing phytosterols on the fermentation characteristic of faeces in perinatal cows

Items	Phytosterols added (mg/d)		SEM	p-value

	0	200		
pH	7.02	7.21	0.120	0.321
NH_3_-N (mg/g)	0.413	0.375	0.067	0.706
MCP (mg/g)	0.440	0.462	0.037	0.686
Total VFA (mmol/g)	0.157	0.154	0.016	0.910
The molar percentage of individual VFA (%)				
Acetate	65.4	67.8	2.88	0.632
Propionate	17.7	16.5	1.48	0.496
Butyrate	13.6	12.4	1.74	0.649
Isobutyrate	1.11	0.89	0.266	0.525
Valerate	1.23	1.24	0.186	0.940
Isovalerate	0.96	1.17	0.145	0.367
Acetate/propionate ratio	3.79	4.45	0.511	0.363

All data were listed as least-squares mean±pooled standard error of means (SEM).

MCP, microbial crude protein; VFA, volatile fatty acid.

The data were deemed significant if p<0.05 and as tendencies, if 0.05< p<0.10.

**Table 6:** Differential metabolites in the faeces of perinatal cows fed with/without phytosterols

Compound	Similarity	Mass	VIP^1)^	FC^2)^	p-value
Isoleucine	315.9	174	1.67	3.19	0.026
4-hydroxybutyrate	388.2	103	1.67	3.19	0.038
2-aminoethanethiol	596.6	174	2.03	2.94	0.004
Tyramine	790.5	174	1.70	2.61	0.023
Phenylethylamine	841.4	174	1.87	2.53	0.010
Hydrocinnamic acid	932.7	104	1.66	2.52	0.025
Linoleic acid	864.5	81	2.21	2.29	0.001
Glutaric acid	834.9	147	1.72	1.86	0.019
Epigallocatechin	377.7	81	1.63	1.84	0.040
Cis-gondoic acid	312.1	131	1.97	1.79	0.006
24,25-dihydrolanosterol	676.2	81	1.64	1.64	0.043
Phytol	941.8	143	1.63	1.57	0.040
Glycine	818.0	174	1.65	1.39	0.043
Shikimic acid	265.8	97	1.81	1.38	0.019
Androsterone	482.5	93	1.51	0.64	0.043

1) Variable importance in projection (VIP) was obtained from orthogonal partial least-squares discrimination analysis of metabolites.

2) Fold change (FC) was calculated through the peak area of each metabolite in the treatment group divided by the peak area of the corresponding metabolite in the control group.

## Data Availability

Raw sequencing reads of bacterial 16S rRNA gene of faeces samples are deposited in the NCBI Sequence Read Archive database (BioProject accession number PRJNA1014226).
